# Elevated platelet-to-lymphocyte ratio is associated with stroke-associated pneumonia in acute ischemic stroke: a retrospective cohort study

**DOI:** 10.3389/fneur.2026.1891885

**Published:** 2026-10-02

**Authors:** Tingting Duan, Chengfei Xu, Jianmei Zhan, Lanzhen Wang, Zhaoping Wu, Yueqi Xu, Junnan Ye, Ming Yang

**Affiliations:** 1 Department of Neurology, The Quzhou Affiliated Hospital of Wenzhou Medical University, Quzhou People’s Hospital, Quzhou, China; 2 Department of Emergency, The Quzhou Affiliated Hospital of Wenzhou Medical University, Quzhou People’s Hospital, Quzhou, China

**Keywords:** stroke-associated pneumonia, platelet-to-lymphocyte ratio, inflammatory biomarkers, risk stratification, non-linear modeling

## Abstract

**Background:**

Stroke-associated pneumonia (SAP) is a common and serious complication of acute ischemic stroke (AIS) that substantially worsens clinical outcomes. The platelet-to-lymphocyte ratio (PLR), an accessible composite index derived from routine complete blood counts, may reflect post-stroke immune imbalance. However, its role in predicting SAP in AIS patients remains unexplored.

**Methods:**

This retrospective cohort study included 1,745 patients with acute ischemic stroke (AIS) after systematic screening of 2,150 patients. After the original screening, 1,766 eligible patients remained; 21 additional patients with extreme PLR values (>1,000) were excluded. HbA1c missing values were imputed once using multiple imputation by chained equations (MICE) with a random forest estimator. PLR was calculated as platelet count divided by lymphocyte count and log2-transformed. Multivariable logistic regression, generalized additive modeling (GAM), and two-piecewise logistic regression were used to examine associations between PLR and stroke-associated pneumonia (SAP). Subgroup analyses were performed across seven clinical variables with interaction tested by likelihood ratio test. Predictive performance was evaluated by receiver operating characteristic (ROC) analysis and compared with C-reactive protein (CRP) and A2DS2 score using the DeLong test.

**Results:**

SAP occurred in 359 patients (20.6%). PLR was higher in patients with SAP than in those without SAP (median 136.17 vs. 113.69, *p* < 0.001). In the fully adjusted model, log2-PLR remained associated with SAP (OR = 1.36 per 1-unit increase, 95% CI: 1.12–1.65, *p* = 0.0021). Compared with the lowest quartile, the highest PLR quartile was associated with increased SAP odds (OR = 1.62, 95% CI: 1.03–2.54, *p* = 0.0386). GAM and two-piecewise regression identified a threshold at PLR = 224.8 (log2-PLR = 7.812). Below this threshold, the association was not significant (OR = 1.21, 95% CI: 0.93–1.57, *p* = 0.1551), whereas above it the association was significant (OR = 2.06, 95% CI: 1.10–3.87, *p* = 0.0240). The AUCs were 0.584 for PLR, 0.825 for CRP, and 0.757 for A2DS2. The AUCs of the PLR + CRP and PLR + A2DS2 models were 0.803 and 0.763, respectively.

**Conclusion:**

Elevated PLR is associated with SAP in AIS patients, exhibiting a non-linear threshold-dependent relationship. Although standalone discriminatory performance is modest, PLR is universally available from routine blood counts and may serve as a cost-effective tool for early SAP risk stratification. Prospective multicenter validation is warranted.

## 1 Introduction

Stroke remains one of the leading causes of mortality and long-term disability worldwide, with acute ischemic stroke (AIS) accounting for approximately 70% of all cases (1–3). Despite substantial advances in acute stroke management, in-hospital complications continue to pose major challenges. Among these, stroke-associated pneumonia (SAP) is particularly consequential, occurring in 10–20% of AIS patients and associated with prolonged hospital stays, escalating healthcare costs, and markedly worse functional outcomes including higher mortality (4–6). Early identification of patients at high risk for SAP remains a clinical priority, as timely preventive interventions may mitigate its occurrence and downstream consequences (5).

Emerging evidence highlights the central role of systemic inflammation in SAP pathogenesis. Stroke triggers a complex post-injury immune response characterized by both paradoxical systemic inflammation and concurrent immunodepression — collectively termed stroke-induced immunodepression syndrome (SIDS) — which disrupts mucosal defense mechanisms and enhances susceptibility to pulmonary infections (7–9). Conventional inflammatory biomarkers including C-reactive protein (CRP), white blood cell (WBC) count, and neutrophil-to-lymphocyte ratio (NLR) have been assessed as predictors of SAP, but their performance remains suboptimal, and more accessible composite markers are needed (10, 11).

The platelet-to-lymphocyte ratio (PLR), calculated simply as platelet count divided by lymphocyte count from a routine complete blood count (CBC), has emerged as an accessible composite index of systemic immune-inflammatory imbalance. PLR integrates two complementary pathophysiological signals relevant to post-stroke susceptibility to infection: thrombocytosis reflects platelet-mediated inflammation and coagulation activation, while lymphopenia indicates suppression of adaptive immunity (12, 13). Elevated PLR has been associated with adverse outcomes in cardiovascular disease, malignancy, and sepsis (14–16). However, whether elevated PLR specifically predicts SAP in AIS patients has not been systematically investigated, and potential non-linear threshold effects have not been explored.

In this study, we aimed to: (1) examine the independent association between admission PLR and SAP occurrence in a large, well-characterized AIS cohort using hierarchical multivariable logistic regression; (2) characterize potential non-linear and threshold-dependent relationships using GAM and two-piecewise regression; (3) assess the robustness of this association across predefined clinical subgroups; and (4) evaluate the predictive performance of PLR and compare it with CRP and A2DS2 score.

## 2 Methods

### 2.1 Study design and participants

This single-center retrospective cohort study was conducted at The Quzhou Affiliated Hospital of Wenzhou Medical University, a 2,000-bed tertiary care and comprehensive stroke center. The study was approved by the hospital’s Institutional Ethics Committee (Approval No. 2023–151) and conducted in accordance with the Declaration of Helsinki. Given its retrospective design and use of de-identified data, the requirement for informed consent was waived. A total of 2,150 consecutive adult patients (aged > = 18 years) admitted with a confirmed diagnosis of AIS between January 2019 and December 2023 were initially screened. The original screening excluded 384 patients: incomplete medical records (*n* = 112), missing laboratory data (*n* = 69), loss to follow-up (*n* = 35), hemorrhagic transformation (*n* = 45), pre-existing pneumonia (*n* = 68), malignancy (*n* = 35), or severe hepatic/renal dysfunction (*n* = 20). After this screening, 1,766 patients were eligible. Loss to follow-up was defined as discharge or transfer to another hospital after which subsequent outcome information could not be obtained because the patient could not be contacted. PLR was then calculated and 21 patients with extreme PLR values >1,000 were excluded, yielding a final analytic cohort of 1,745 patients (Figure 1). Because some exclusion reasons may overlap, the reason-specific counts are reported separately from the total number of excluded patients. AIS was diagnosed by a senior neurologist based on clinical presentation and confirmed by computed tomography (CT) or magnetic resonance imaging (MRI).

**Figure 1:**
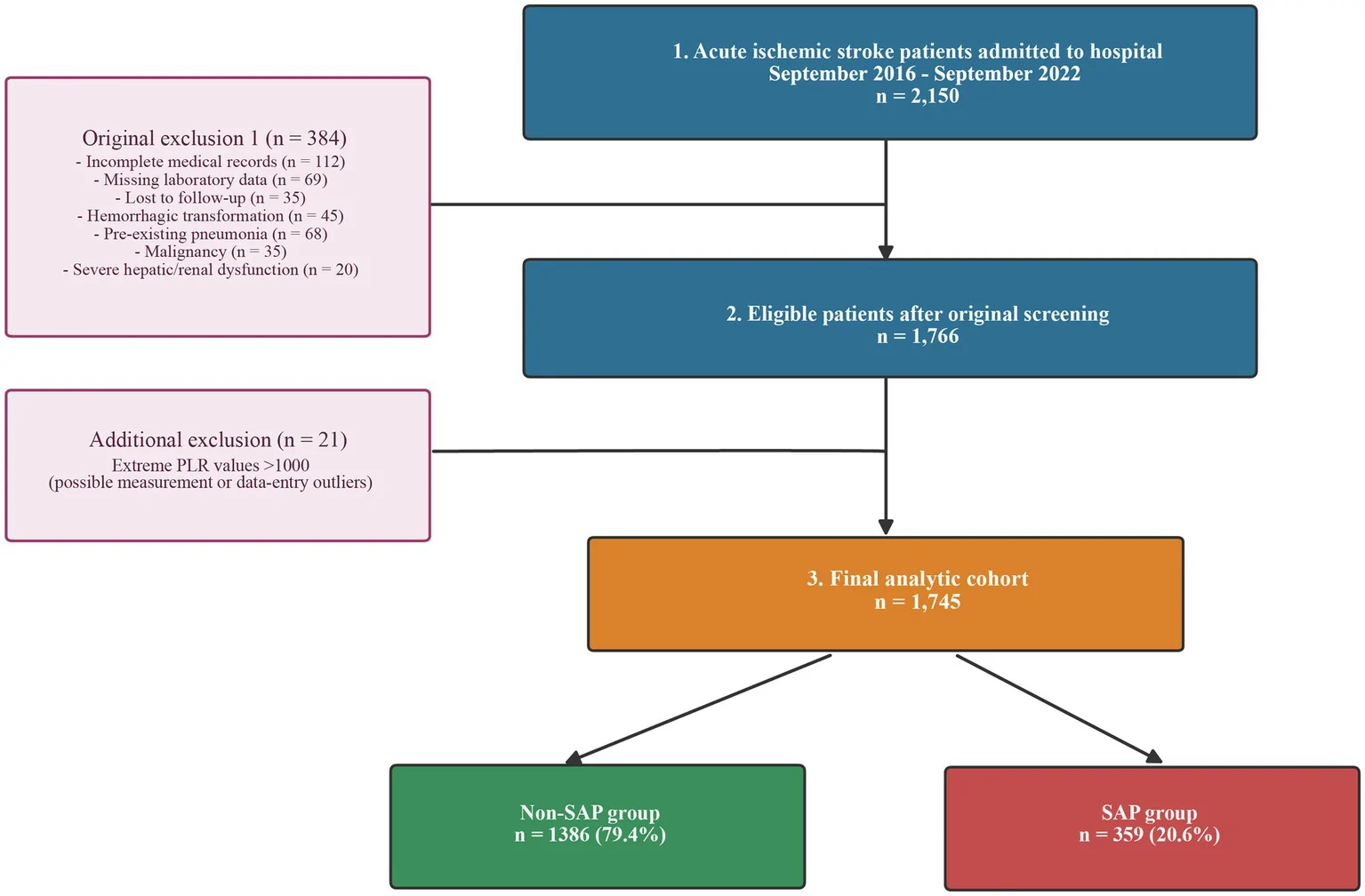
Patient selection flowchart demonstrating systematic derivation of the analytic cohort. A total of 2,150 patients with acute ischemic stroke admitted between January 2019 and December 2023 were screened. The original screening excluded 384 patients: incomplete medical records (*n* = 112), missing laboratory data (*n* = 69), loss to follow-up (*n* = 35), hemorrhagic transformation (*n* = 45), pre-existing pneumonia (*n* = 68), malignancy (*n* = 35), and severe hepatic/renal dysfunction (*n* = 20). After original screening, 1,766 patients remained. An additional 21 patients with extreme PLR values >1,000 were excluded during PLR calculation and extreme-value screening, leaving 1,745 patients in the final analytic cohort; 1,386 were non-SAP and 359 were SAP. Loss to follow-up refers to discharge or transfer after which subsequent outcome information could not be obtained because the patient could not be contacted. Reason-specific exclusion counts may overlap. SAP, stroke-associated pneumonia; PLR, platelet-to-lymphocyte ratio.

### 2.2 Definition of stroke-associated pneumonia

SAP was defined according to the revised standards of the Centers for Disease Control and Prevention and the pneumonia recommendations of the Stroke Consensus Group (17). The observation window extended from hospital admission to discharge or in-hospital death. SAP required (1) a new or progressive pulmonary infiltrate on chest radiography or CT and (2) at least two of the following: fever (>38.0 degrees C) or hypothermia (<36.0 degrees C), leukocytosis (>11 × 10^9/L) or leukopenia (<4 × 10^9/L), purulent respiratory secretions, or newly developed cough or dyspnea. Two attending neurologists independently confirmed each SAP diagnosis; disagreements were resolved by a third neurologist. Patients with pneumonia present at admission were excluded, so only incident in-hospital SAP was captured.

### 2.3 PLR calculation and covariate assessment

Venous blood samples were obtained within 24 h of admission. PLR was calculated as: PLR = platelet count (x 10^9/L) / lymphocyte count (x 10^9/L). PLR values were log2-transformed before regression to reduce right skewness and facilitate per-unit-increase interpretation. One patient with missing eGFR was removed during data cleaning. Missing HbA1c values were imputed once using MICE with a random forest estimator; no multiple-imputation pooling was performed. After PLR calculation, values >1,000 were excluded as extreme values potentially reflecting measurement or data-entry outliers. Covariates recorded at admission included: age, sex, smoking status, hypertension, diabetes mellitus, atrial fibrillation (AF), COPD, fasting plasma glucose (FPG), glycated hemoglobin (HbA1c), triglycerides (TG), total cholesterol (TC), uric acid (UA), homocysteine (HCY), CRP, WBC, albumin, estimated glomerular filtration rate (eGFR; CKD-EPI formula) (18), National Institutes of Health Stroke Scale (NIHSS) score, Kubota Water Drinking Test (KWDT) result, and A2DS2 score (19). To assess robustness of the extreme-value rule, we repeated the fully adjusted analysis using the imputed dataset before PLR extreme-value exclusion and performed leave-one-extreme-observation-out influence diagnostics; the results are reported in Section 3.5.

### 2.4 Multicollinearity assessment and covariate selection

Multicollinearity was assessed using variance inflation factors (VIF). All 26 candidate covariates were examined; VIF > =10 was considered severe collinearity. The maximum VIF was 8.726 for neutrophil count (NTC), and all VIF values were below 10. LDL-C was excluded from the regression models because of its collinearity with TC (VIF = 5.943). NTC was excluded as a direct structural component of PLR and a potential pathway mediator. A2DS2 was excluded from the regression models because of collinearity with NIHSS and KWDT (VIF = 7.688).

### 2.5 Statistical analysis

Continuous variables are expressed as mean ± SD (normally distributed; assessed by Kolmogorov–Smirnov test) or median (IQR) (non-normally distributed). Between-group comparisons used Student’s t-test or Mann–Whitney U test for continuous variables, and chi-square test for categorical variables.

Three hierarchical logistic regression models were constructed: Model 1 (unadjusted); Model 2 (adjusted for age and sex); and Model 3 (fully adjusted for age, sex, smoking, hypertension, diabetes, AF, COPD, FPG, HbA1c, TG, TC, UA, HCY, CRP, WBC, albumin, eGFR, NIHSS, and KWDT). PLR was analyzed both as a continuous variable (log2-transformed, per 1-unit increase) and categorized into quartiles. *p* for trend was calculated by entering quartile number (1–4) as a continuous ordinal variable. To assess multicollinearity among covariates in the multivariable model, VIFs were calculated; all VIF values were below 10, indicating no severe collinearity (Supplementary Table S1).

Non-linear associations were explored using GAM with penalized splines. Two-piecewise logistic regression was applied to identify an inflection point (K), selected as the cut-point within the 10th-90th percentile range yielding the maximum log-likelihood; the 95% CI of K was estimated by 200 bootstrap resamples. A log-likelihood ratio test compared the piecewise model with the linear model.

Subgroup analyses were performed for seven pre-specified binary variables: age (<70 vs. ≥ 70 years), sex, smoking, hypertension, diabetes, AF, and COPD. In each stratum, a model adjusted for all Model 3 covariates excluding the stratifying variable was fitted. Interaction was assessed by likelihood ratio test for a multiplicative PLR × subgroup term.

ROC analysis was performed in the final analytic cohort (*N* = 1,745; SAP = 359). AUC with 95% CI was estimated by the DeLong method. PLR was compared individually with CRP and A2DS2; combined logistic models (PLR + CRP; PLR + A2DS2) were also evaluated. AUC comparisons used the DeLong test. Sensitivity, specificity, and accuracy at the Youden-optimal cut-off are reported. All analyses were performed using Python (version 3.11.1). Two-sided *p* < 0.05 was considered statistically significant. Internal validation of Model 3 was performed using 10-fold out-of-fold cross-validation and 1,000 bootstrap resamples. Calibration intercept and slope, Brier score, log loss, and decision-curve net benefit were evaluated from out-of-fold predictions. External validation was not performed because an independent cohort was unavailable.

## 3 Results

### 3.1 Baseline characteristics by SAP status

Among 1,745 AIS patients, SAP developed in 359 (20.6%) during hospitalization. The mean age was 69.31 ± 12.40 years, and 1,035 patients (59.3%) were male. Compared with non-SAP patients, those with SAP were significantly older (74.87 ± 11.35 vs. 67.87 ± 12.26 years, *p* < 0.001), and more frequently had atrial fibrillation (35.1% vs. 11.5%), COPD (15.3% vs. 4.0%), nasal feeding (37.3% vs. 2.5%), and disturbance of consciousness (25.1% vs. 2.0%, all *p* < 0.001). Sex, smoking status, hypertension, and diabetes did not differ significantly between groups.

Laboratory findings showed higher PLR in SAP patients than in non-SAP patients (136.17 [IQR 89.50–201.95] vs. 113.69 [84.31–157.85], *p* < 0.001), together with markedly higher CRP (13.90 vs. 2.00 mg/L) and WBC (9.87 ± 3.77 vs. 6.84 ± 2.09 × 10^9/L, both *p* < 0.001). SAP patients also had higher FPG, HCY, and NTC, and lower TG, TC, UA, albumin, and eGFR (all *p* < 0.05). Neurological severity was greater in the SAP group, reflected by higher NIHSS (median 5 vs. 2), higher A2DS2 (4 vs. 2), higher KWDT, and lower GCS scores (all *p* < 0.001). Detailed baseline characteristics are presented in Table 1.

**Table 1:** Baseline characteristics of patients with acute ischemic stroke, stratified by stroke-associated pneumonia (SAP) status.

Variables	Overall (*N* = 1745)	Non-SAP (*N* = 1,386)	SAP (*N* = 359)	*p*-value
*N*	1745	1,386	359	
Age (years)	69.31 ± 12.40	67.87 ± 12.26	74.87 ± 11.35	**<0.001**
Temperature (°C)	36.50 ± 0.47	36.49 ± 0.37	36.53 ± 0.72	0.166
Gender, *n* (%)				
Male	1,035 (59.3%)	835 (60.2%)	200 (55.7%)	
Female	710 (40.7%)	551 (39.8%)	159 (44.3%)	
Smoke, *n* (%)	637 (36.5%)	507 (36.6%)	130 (36.2%)	
Hypertension, *n* (%)	1,341 (76.8%)	1,066 (76.9%)	275 (76.6%)	
Diabetes, *n* (%)	622 (35.6%)	496 (35.8%)	126 (35.1%)	
Atrial fibrillation, *n* (%)	286 (16.4%)	160 (11.5%)	126 (35.1%)	
Nasal feeding, *n* (%)	168 (9.6%)	34 (2.5%)	134 (37.3%)	
Disturbance of consciousness, *n* (%)	118 (6.8%)	28 (2.0%)	90 (25.1%)	
COPD, *n* (%)	111 (6.4%)	56 (4.0%)	55 (15.3%)	
Laboratory parameters				
PLT (×10^9^/L)	188.00 (151.00, 231.00)	188.00 (152.00, 229.00)	188.00 (146.50, 240.50)	0.650
PLR	117.09 (85.09, 164.48)	113.69 (84.31, 157.85)	136.17 (89.50, 201.95)	**<0.001**
FPG (mg/dL)	101.63 (89.92, 127.94)	100.01 (89.42, 124.65)	108.12 (92.80, 138.66)	**<0.001**
HbA1c (%)	6.00 (5.52, 7.10)	6.00 (5.50, 7.10)	6.00 (5.60, 7.15)	0.386
TG (mg/dL)	113.37 (80.60, 155.00)	117.80 (84.14, 163.85)	93.00 (69.08, 127.54)	**<0.001**
TC (mmol/L)	4.27 (3.65, 5.01)	4.30 (3.69, 5.03)	4.17 (3.55, 4.94)	0.020
LDL-C (mmol/L)	2.78 (2.18, 3.41)	2.80 (2.21, 3.42)	2.70 (2.06, 3.33)	0.109
UA (μmol/L)	311.70 (251.00, 379.10)	313.00 (257.10, 381.98)	302.70 (235.70, 367.05)	0.020
HCY (μmol/L)	14.92 (11.70, 19.20)	14.60 (11.50, 19.00)	16.30 (12.90, 20.10)	**<0.001**
CRP (mg/L)	2.80 (1.13, 6.62)	2.00 (1.00, 4.26)	13.90 (4.00, 37.13)	**<0.001**
WBC (×10^9^/L)	7.46 ± 2.81	6.84 ± 2.09	9.87 ± 3.77	**<0.001**
NTC (×10^9^/L)	4.51 (3.48, 6.10)	4.20 (3.30, 5.30)	6.87 (5.12, 9.01)	**<0.001**
Albumin (g/L)	37.97 ± 3.83	38.42 ± 3.57	36.22 ± 4.30	**<0.001**
eGFR (mL/min/1.73 m^2^)	99.29 (79.56, 118.81)	100.60 (81.74, 119.32)	92.45 (71.03, 115.65)	**<0.001**
Clinical characteristics				
NIHSS	3.00 (1.00, 6.00)	2.00 (1.00, 5.00)	5.00 (2.00, 13.00)	**<0.001**
KWDT	1.00 (1.00, 1.00)	1.00 (1.00, 1.00)	1.00 (1.00, 4.00)	**<0.001**
A2DS2	3.00 (2.00, 4.00)	2.00 (2.00, 4.00)	4.00 (3.00, 6.50)	**<0.001**
GCS	15.00 (14.00, 15.00)	15.00 (15.00, 15.00)	14.00 (12.00, 15.00)	**<0.001**

Values are presented as mean ± standard deviation for normally distributed continuous variables, median (interquartile range) for non-normally distributed continuous variables, or *n* (%) for categorical variables. HbA1c missing values were imputed once using MICE with a random forest estimator. The analytic cohort excluded one patient with missing eGFR and 21 patients with PLR >1,000.

*p*-values were calculated using Student’s *t*-test or Mann–Whitney U test for continuous variables and chi-square test for categorical variables.

SAP, stroke-associated pneumonia; PLT, platelet count; PLR, platelet-to-lymphocyte ratio; COPD, chronic obstructive pulmonary disease; FPG, fasting plasma glucose; HbA1C, glycated hemoglobin; TG, triglycerides; TC, total cholesterol; LDL-C, low-density lipoprotein cholesterol; UA, uric acid; HCY, homocysteine; CRP, C-reactive protein; WBC, white blood cell count; NTC, neutrophil count; eGFR, estimated glomerular filtration rate; NIHSS, National Institutes of Health Stroke Scale; KWDT, Kubota Water Drinking Test; A2DS2, age, atrial fibrillation, dysphagia, sex, stroke severity; GCS, Glasgow Coma Scale.

Bold *p*-values indicate statistical significance (*p* < 0.05).

### 3.2 Baseline characteristics by PLR quartile

PLR quartile boundaries were: Q1 (5.8–85.1), Q2 (85.1–117.1), Q3 (117.1–164.5), and Q4 (164.9–973.5). SAP incidence was 17.6% in Q1, 15.8% in Q2, 19.5% in Q3, and 29.4% in Q4 (*p* < 0.001). The proportion of male patients decreased across quartiles, whereas AF, COPD, CRP, and NTC tended to be higher in the upper quartiles. Full characteristics by PLR quartile are shown in Table 2.

**Table 2:** Baseline characteristics of patients stratified by quartiles of platelet-to-lymphocyte ratio (PLR).

Variables	Q1 (*N* = 437) *PLR: 5.8–85.1*	Q2 (*N* = 436) *PLR: 85.1–117.1*	Q3 (*N* = 436) *PLR: 117.1–164.5*	Q4 (*N* = 436) *PLR: 164.9–973.5*	*p*-value
*N*	437	436	436	436	
SAP, *n* (%)	77 (17.6%)	69 (15.8%)	85 (19.5%)	128 (29.4%)	**<0.001**
Age (years)	68.24 ± 12.59	69.20 ± 12.64	69.47 ± 12.59	70.34 ± 11.71	0.095
Temperature (°C)	36.44 ± 0.62	36.51 ± 0.39	36.52 ± 0.39	36.55 ± 0.42	**0.005**
Gender, *n* (%)					
Male	285 (65.2%)	264 (60.6%)	264 (60.6%)	222 (50.9%)	**<0.001**
Female	152 (34.8%)	172 (39.4%)	172 (39.4%)	214 (49.1%)	
Smoke, *n* (%)	189 (43.2%)	160 (36.7%)	154 (35.3%)	134 (30.7%)	**0.002**
Hypertension, *n* (%)	331 (75.7%)	350 (80.3%)	331 (75.9%)	329 (75.5%)	0.277
Diabetes, *n* (%)	186 (42.6%)	143 (32.8%)	148 (33.9%)	145 (33.3%)	**0.006**
Atrial fibrillation, *n* (%)	61 (14.0%)	60 (13.8%)	85 (19.5%)	80 (18.3%)	**0.039**
Nasal feeding, *n* (%)	24 (5.5%)	32 (7.3%)	46 (10.6%)	66 (15.1%)	**<0.001**
Disturbance of consciousness, *n* (%)	25 (5.7%)	21 (4.8%)	28 (6.4%)	44 (10.1%)	**0.011**
COPD, *n* (%)	20 (4.6%)	25 (5.7%)	26 (6.0%)	40 (9.2%)	**0.036**
Laboratory parameters					
PLT (×10^9^/L)	155.00 (127.00–195.00)	186.00 (150.75–222.00)	197.00 (164.75–237.25)	222.00 (175.00–270.00)	**<0.001**
PLR	68.45 (57.63–77.30)	100.69 (93.96–107.98)	136.17 (126.87–149.34)	219.02 (183.63–283.90)	**<0.001**
FPG (mg/dL)	103.43 (90.46–131.55)	98.75 (89.56–124.74)	101.00 (89.78–127.99)	101.99 (90.10–129.61)	0.368
HbA1c (%)	6.20 (5.60–7.50)	5.97 (5.59–6.96)	6.00 (5.51–6.92)	5.90 (5.50–7.10)	**0.047**
TG (mg/dL)	119.57 (85.03–170.94)	120.01 (85.91–173.38)	109.38 (77.06–146.36)	101.86 (74.18–136.62)	**<0.001**
TC (mmol/L)	4.22 (3.63–4.89)	4.29 (3.65–5.11)	4.24 (3.61–5.06)	4.33 (3.69–4.96)	0.569
LDL-C (mmol/L)	2.77 (2.20–3.33)	2.75 (2.12–3.50)	2.80 (2.11–3.39)	2.80 (2.24–3.40)	0.887
UA (μmol/L)	331.00 (268.50–391.50)	312.15 (255.78–382.60)	309.50 (251.90–368.43)	294.80 (236.60–366.35)	**<0.001**
HCY (μmol/L)	14.70 (12.08–19.30)	15.05 (11.67–19.52)	15.30 (11.67–19.92)	14.60 (11.58–18.29)	0.353
CRP (mg/L)	2.24 (1.03–5.13)	2.20 (1.00–5.54)	3.00 (1.23–7.78)	3.46 (1.40–10.96)	**<0.001**
WBC (×10^9^/L)	7.84 ± 2.83	7.30 ± 2.63	7.36 ± 2.89	7.35 ± 2.84	**0.013**
NTC (×10^9^/L)	4.21 (3.39–5.42)	4.31 (3.40–5.89)	4.66 (3.58–6.10)	5.03 (3.69–6.88)	**<0.001**
Albumin (g/L)	38.13 ± 3.59	38.21 ± 4.07	37.68 ± 3.75	37.85 ± 3.89	0.141
eGFR (mL/min/1.73 m^2^)	98.93 (79.50–119.07)	97.75 (80.86–117.48)	98.85 (77.72–117.49)	102.87 (82.00–121.33)	0.374
Clinical characteristics					
NIHSS	2.00 (1.00–5.00)	2.00 (1.00–5.00)	3.00 (1.00–6.00)	3.00 (2.00–7.00)	**<0.001**
KWDT	1.00 (1.00–1.00)	1.00 (1.00–1.00)	1.00 (1.00–1.00)	1.00 (1.00–1.00)	**<0.001**
A2DS2	2.00 (2.00–4.00)	2.00 (2.00–4.00)	3.00 (2.00–4.00)	3.00 (2.00–4.00)	**0.001**
GCS	15.00 (14.00–15.00)	15.00 (15.00–15.00)	15.00 (14.00–15.00)	15.00 (13.00–15.00)	**<0.001**

Values are presented as mean ± standard deviation for normally distributed continuous variables, median (interquartile range) for non-normally distributed continuous variables, or *n* (%) for categorical variables. HbA1c missing values were imputed once using MICE with a random forest estimator. PLR ranges are observed minimum-to-maximum values within each quartile; rounding may produce small overlaps or gaps between displayed ranges.

*p*-values for continuous variables were calculated using one-way ANOVA or Kruskal-Wallis test; for categorical variables, chi-square test was used.

SAP, stroke-associated pneumonia; PLT, platelet count; PLR, platelet-to-lymphocyte ratio; COPD, chronic obstructive pulmonary disease; FPG, fasting plasma glucose; HbA1C, glycated hemoglobin; TG, triglycerides; TC, total cholesterol; LDL-C, low-density lipoprotein cholesterol; UA, uric acid; HCY, homocysteine; CRP, C-reactive protein; WBC, white blood cell count; NTC, neutrophil count; eGFR, estimated glomerular filtration rate; NIHSS, National Institutes of Health Stroke Scale; KWDT, Kubota Water Drinking Test; A2DS2, age, atrial fibrillation, dysphagia, sex, stroke severity; GCS, Glasgow Coma Scale.

Bold *p*-values indicate statistical significance (*p* < 0.05).

### 3.3 Association between PLR and SAP: multivariable logistic regression

Results of the logistic regression analyses are presented in Table 3. In the unadjusted model (Model 1), each 1-unit increase in log2-PLR was associated with higher odds of SAP (OR = 1.50, 95% CI: 1.30–1.74, *p* < 0.001). After adjustment for age and sex (Model 2), the association remained significant (OR = 1.47, 95% CI: 1.27–1.71, *p* < 0.001). In the fully adjusted model (Model 3), log2-PLR remained associated with SAP (OR = 1.36, 95% CI: 1.12–1.65, *p* = 0.0021).

**Table 3:** Multivariable logistic regression analysis of the association between log_2_-transformed PLR and stroke-associated pneumonia (SAP).

Exposure	Crude model (Model 1) OR (95% CI)	*p*-value	Partially adjusted (Model 2) OR (95% CI)	*p*-value	Fully adjusted (Model 3) OR (95% CI)	*p*-value
log₂-PLR (per 1-unit increase)						
PLR (continuous) *N* = 1745	**1.50 (1.30–1.74)**	**<0.001**	**1.47 (1.27–1.71)**	**<0.001**	**1.36 (1.12–1.65)**	**0.0021**
PLR quartile						
Q1 (PLR: 5.8–85.1) *N* = 437, SAP = 77 (17.6%)	1.00 (Reference)		1.00 (Reference)		1.00 (Reference)	
Q2 (PLR: 85.1–117.1) *N* = 436, SAP = 69 (15.8%)	0.88 (0.62–1.25)	0.4777	0.83 (0.58–1.20)	0.3224	0.86 (0.53–1.40)	0.5422
Q3 (PLR: 117.1–164.5) *N* = 436, SAP = 85 (19.5%)	1.13 (0.80–1.59)	0.4762	1.07 (0.75–1.52)	0.6971	0.91 (0.57–1.45)	0.6842
Q4 (PLR: 164.9–973.5) *N* = 436, SAP = 128 (29.4%)	**1.94 (1.41–2.68)**	**<0.001**	**1.86 (1.33–2.59)**	**<0.001**	**1.62 (1.03–2.54)**	**0.0386**
*p* for trend		**<0.001**		**<0.001**		**0.0351**

OR, odds ratio; CI, confidence interval; PLR, platelet-to-lymphocyte ratio; SAP, stroke-associated pneumonia.

Model 1: Unadjusted (crude model).

Model 2: Adjusted for age and gender.

Model 3: Adjusted for Age, Gender, Smoke, Hypertension, Diabetes, Atrial fibrillation, COPD, FPG, HbA1C, TG, TC, UA, HCY, CRP, WBC, Albumin, eGFR, NIHSS, KWDT.

LDL-C was excluded due to collinearity with TC (VIF = 5.943). NTC was excluded as a direct component of PLR and a potential mediator. A2DS2 was excluded due to collinearity with NIHSS and KWDT (VIF = 7.688).

HbA1c missing values were imputed once using MICE with a random forest estimator; the final analytic sample was *N* = 1,745.

*p* for trend was calculated by entering the quartile number (1–4) as a continuous ordinal variable in the logistic regression model. PLR ranges are observed minimum-to-maximum values within each quartile; rounding may produce small overlaps or gaps between displayed ranges.

Bold OR and *p*-values indicate statistical significance (*p* < 0.05).

FPG, fasting plasma glucose; HbA1C, glycated hemoglobin; TG, triglycerides; TC, total cholesterol; UA, uric acid; HCY, homocysteine; CRP, C-reactive protein; WBC, white blood cell count; eGFR, estimated glomerular filtration rate; NIHSS, National Institutes of Health Stroke Scale; KWDT, Kubota Water Drinking Test; COPD, chronic obstructive pulmonary disease.

In the quartile analysis, compared with Q1 as reference, patients in Q4 had significantly higher SAP odds after full adjustment (OR = 1.62, 95% CI: 1.03–2.54, *p* = 0.0386). Q2 and Q3 were not significantly different from Q1 in Model 3. A significant trend across quartiles was observed in Models 1 and 2 (both *p* < 0.001) and remained significant in Model 3 (*p* for trend = 0.0351).

### 3.4 Non-linear association and threshold effect

GAM analysis revealed a non-linear, threshold-dependent relationship between log2-PLR and SAP probability, with a relatively flat risk at lower PLR values and an increase beyond the inflection point (Figure 2). Two-piecewise logistic regression identified the inflection point at log2-PLR = 7.812 (corresponding to PLR = 224.8). Below this threshold, PLR was not significantly associated with SAP (OR = 1.21, 95% CI: 0.93–1.57, *p* = 0.1551). Above the threshold, the association was significant (OR = 2.06, 95% CI: 1.10–3.87, *p* = 0.0240). The difference between the two segment effects was not statistically significant (OR = 1.71, 95% CI: 0.87–3.38, *p* = 0.1228), and the log-likelihood ratio test was not significant (*p* = 0.1724).

**Figure 2:**
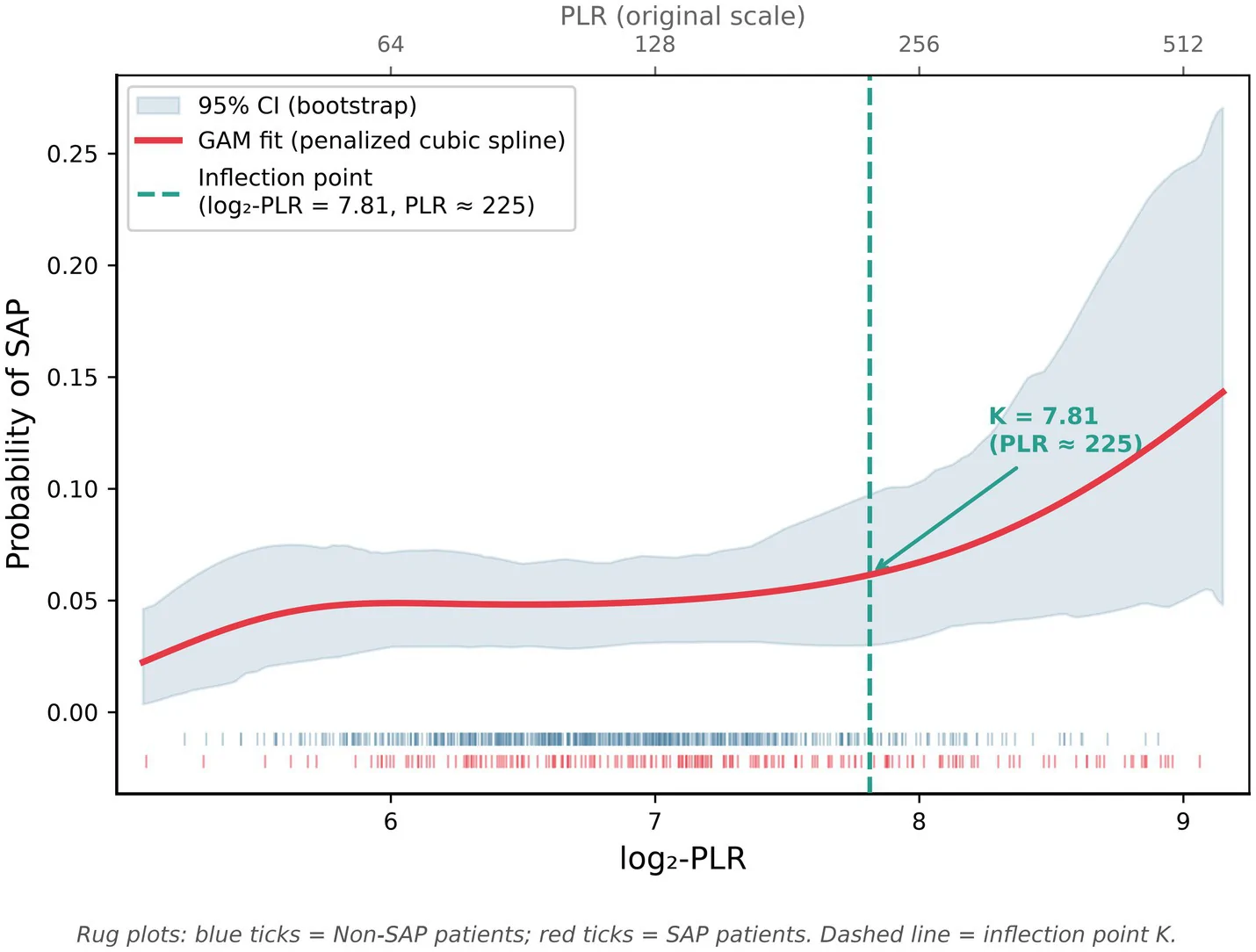
Generalized additive model (GAM) and threshold effect analysis of the association between log₂-PLR and SAP probability. The red curve represents the GAM fit (penalized spline) with 95% confidence interval (shaded blue). The dashed teal vertical line indicates the inflection point identified by two-piecewise logistic regression (*K* = 7.812, corresponding to PLR = 224.8). Rug plots indicate individual patient positions along the log₂-PLR axis: blue ticks, non-SAP patients; red ticks, SAP patients. Both axes are shown: log₂-PLR (lower x-axis) and original PLR scale (upper x-axis). PLR, platelet-to-lymphocyte ratio; SAP, stroke-associated pneumonia; GAM, generalized additive model. Rug plots: blue ticks = Non-SAP patients; red ticks = SAP patients. Dashed line = inflection point K.

### 3.5 Subgroup analyses

Subgroup analyses were conducted across age (<70 vs. > =70 years), sex, smoking status, hypertension, diabetes, AF, and COPD (Figure 3). The association between elevated PLR and SAP was generally consistent across subgroups, with adjusted ORs ranging from 1.07 (95% CI: 0.68–1.67) in patients without hypertension to 2.25 (95% CI: 0.71–7.14) in patients with COPD. No significant interaction between PLR and any subgroup variable was detected (all *p* for interaction > 0.05), supporting the robustness of the association.

**Figure 3:**
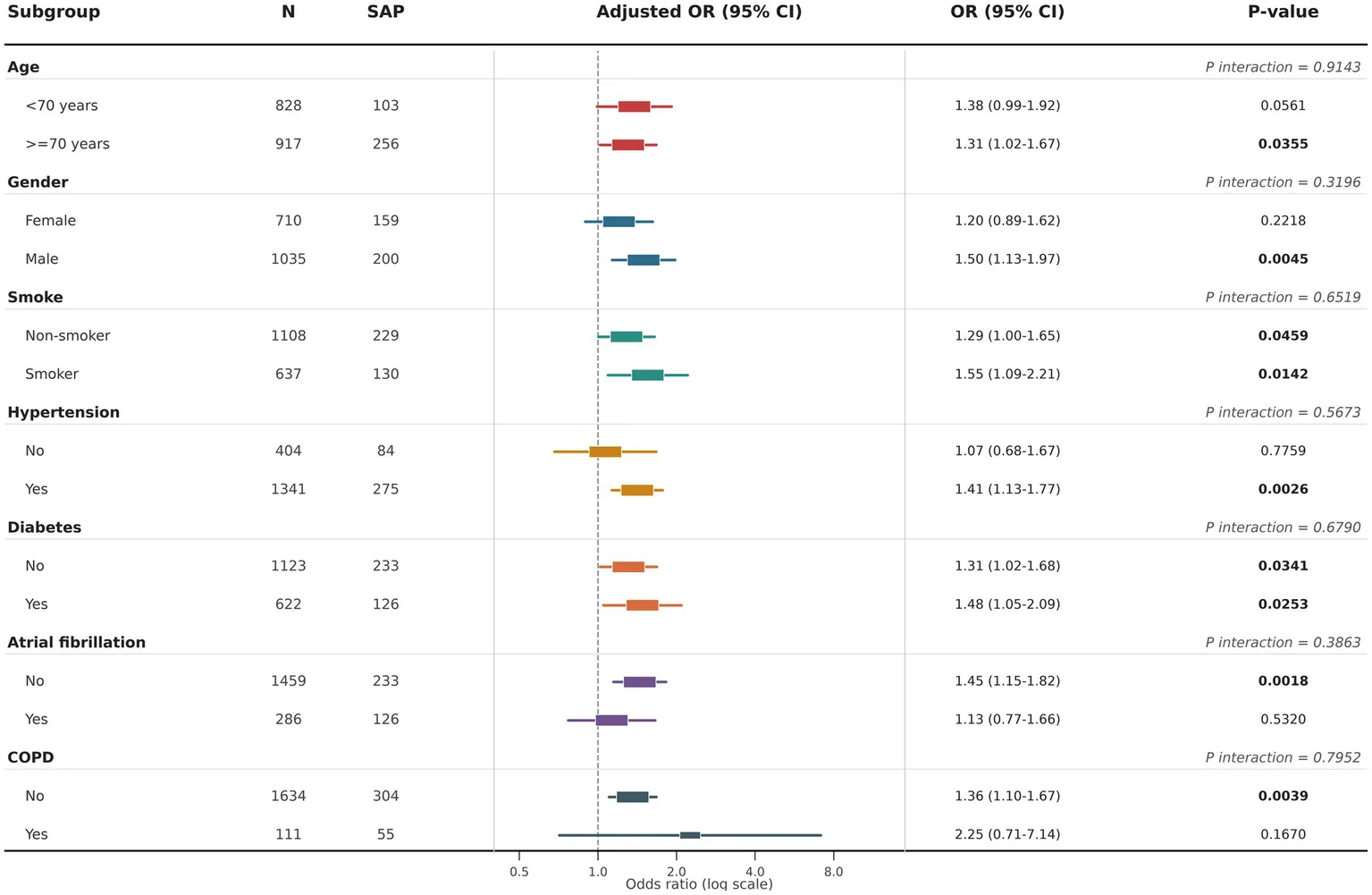
Subgroup analysis of the association between PLR and SAP. Forest plot illustrating adjusted odds ratios (ORs) and 95% confidence intervals (CIs) for the association between log2-PLR and SAP across seven predefined subgroups in the final analytic cohort. Each stratum was adjusted for all Model 3 covariates excluding the stratifying variable. Interaction *p*-values were derived from the likelihood ratio test for the multiplicative PLR x subgroup interaction term. All *p* for interaction > 0.05. PLR, platelet-to-lymphocyte ratio; SAP, stroke-associated pneumonia; OR, odds ratio; CI, confidence interval; COPD, chronic obstructive pulmonary disease.

### 3.6 Sensitivity analysis of extreme PLR values

To evaluate the effect of extreme PLR observations, we repeated the analysis in the pre-exclusion imputed dataset (*N* = 1,766; SAP = 376), which retained 21 PLR values >1,000, and compared it with the primary cohort after their exclusion (*N* = 1,745; SAP = 359). In the pre-exclusion dataset, the fully adjusted association for continuous log2-PLR was OR = 1.45 (95% CI: 1.23–1.71; *p* < 0.001), compared with OR = 1.36 (95% CI: 1.12–1.65; *p* = 0.0021) in the primary cohort. PLR AUC was 0.601 (95% CI: 0.566–0.636) before exclusion and 0.584 (95% CI: 0.549–0.619) after exclusion. Leave-one-extreme-observation-out analyses produced Model 3 ORs from 1.421 to 1.480; the maximum Cook distance was 0.0062 and the maximum absolute DFBETA for log2-PLR was 0.247. These findings indicate that the association was not driven by a single extreme observation, although the standalone discriminatory performance of PLR remained modest.

### 3.7 Predictive performance: ROC analysis

ROC analyses were performed in the final analytic cohort (*N* = 1,745, SAP = 359). The AUC of PLR alone for predicting SAP was 0.584 (95% CI: 0.549–0.619), with 49.9% sensitivity and 66.5% specificity at the Youden-optimal cut-off. CRP demonstrated superior discrimination (AUC = 0.825, 95% CI: 0.799–0.851; DeLong *p* < 0.0001 vs. PLR), with 71.3% sensitivity and 81.1% specificity. A2DS2 also outperformed PLR (AUC = 0.757, 95% CI: 0.727–0.786; DeLong *p* < 0.0001 vs. PLR).

The combined model PLR + CRP yielded an AUC of 0.803 (95% CI: 0.775–0.832), which was not significantly different from CRP alone (DeLong *p* = 0.0847). Similarly, PLR + A2DS2 yielded an AUC of 0.763 (95% CI: 0.733–0.793), with no significant difference from A2DS2 alone (*p* = 0.3491). Comprehensive diagnostic metrics are summarized in Table 4, and ROC curves are illustrated in Figure 4. For the fully adjusted Model 3, the apparent AUC was 0.906, the 10-fold out-of-fold AUC was 0.900, and the bootstrap optimism-corrected AUC was 0.897. The apparent and out-of-fold Brier scores were 0.083 and 0.086, respectively; the out-of-fold calibration intercept and slope were −0.068 and 0.942. Out-of-fold decision-curve analysis showed positive net benefit across threshold probabilities of 0.05–0.50 (Supplementary Table S3; Supplementary Figures S1–S3).

**Table 4:** Comparison of diagnostic performance of PLR, CRP, A2DS2, and their combination models for predicting stroke-associated pneumonia.

Model	AUC	95% CI	Sensitivity (%)	Specificity (%)	Accuracy (%)	DeLong *p*-value
PLR	0.584	0.549–0.619	49.9	66.5	63.0	Reference
CRP	0.825	0.799–0.851	71.3	81.1	79.0	vs PLR: <0.0001
A2DS2	0.757	0.727–0.786	48.2	90.5	71.7	vs PLR: <0.0001
PLR + CRP	0.803	0.775–0.832	64.3	85.9	81.4	vs CRP: 0.0847; vs. PLR: <0.0001
PLR + A2DS2	0.763	0.733–0.793	53.8	87.7	80.7	vs A2DS2: 0.3491

Table summarizes the diagnostic performance of log2-transformed PLR, CRP, A2DS2, and their combinations for predicting SAP in the final analytic cohort (*N* = 1,745, SAP = 359). HbA1c missing values were imputed once using MICE with a random forest estimator.

The combined models (PLR + CRP and PLR + A2DS2) were derived from binary logistic regression using the predicted probabilities as the ROC score.

Sensitivity and specificity were determined at the optimal cut-off point using the Youden index (maximum of sensitivity + specificity − 1). Statistical comparisons between AUCs were performed using the DeLong test.

AUC, area under the ROC curve; CI, confidence interval; PLR, platelet-to-lymphocyte ratio; CRP, C-reactive protein; A2DS2, Age, Atrial Fibrillation, Dysphagia, Stroke Severity and Sex; SAP, stroke-associated pneumonia; ROC, receiver operating characteristic.

**Figure 4:**
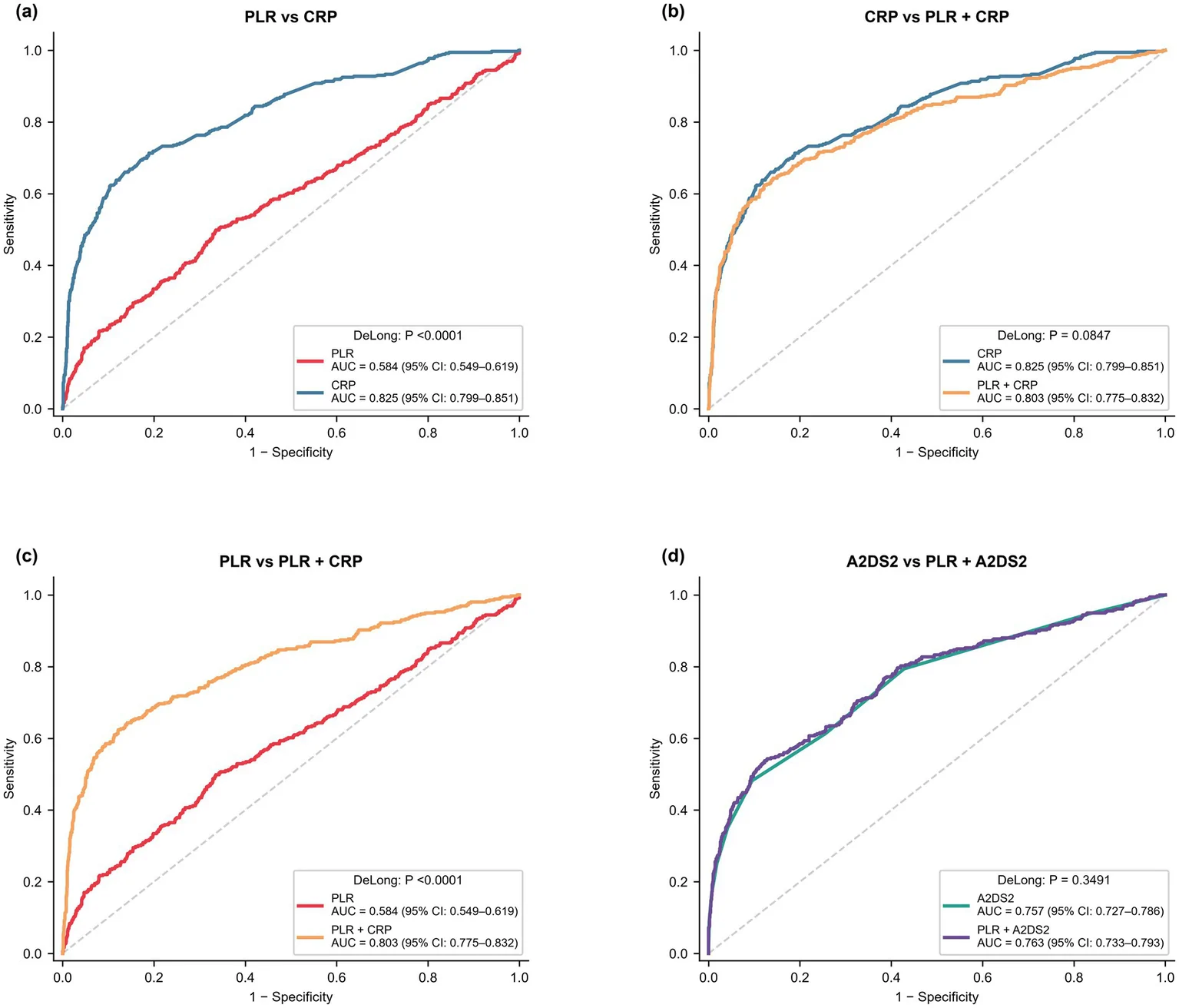
Receiver operating characteristic (ROC) curves for predictive performance of PLR, CRP, A2DS2, and combination models for SAP. **(a)** PLR (*AUC* = 0.584) and CRP (*AUC* = 0.825), with significantly superior discrimination for CRP (DeLong *p* < 0.0001). **(b)** CRP alone versus PLR + CRP (*AUC* = 0.803), with no significant difference (*p* = 0.0847). **(c)** PLR alone versus PLR + CRP, with the combination significantly outperforming PLR alone (*p* < 0.0001). **(d)** A2DS2 (*AUC* = 0.757) versus PLR + A2DS2 (*AUC* = 0.763), with no significant improvement (*p* = 0.3491). AUC, area under the curve; PLR, platelet-to-lymphocyte ratio; CRP, C-reactive protein; A2DS2, Age, Atrial Fibrillation, Dysphagia, Stroke Severity and Sex; SAP, stroke-associated pneumonia.

## 4 Discussion

In this retrospective cohort of 1,745 AIS patients, we demonstrate that elevated admission PLR is associated with SAP risk across all three hierarchical regression models. A non-linear, threshold-dependent relationship was identified at PLR = 224.8, below which no significant association was observed and above which SAP risk increased. This association was consistent across all seven predefined clinical subgroups with no significant interactions.

The biological rationale for a PLR–SAP association is grounded in the dual pathophysiological significance of its components. Platelets are not merely hemostatic effectors; they function as key modulators of innate immunity, releasing P-selectin, platelet factor 4, and interleukin-1β upon activation (20–24). Stroke triggers intense platelet activation through thrombin generation and endothelial injury, contributing to a pro-inflammatory systemic state (24–26). Conversely, post-stroke lymphopenia reflects suppression of adaptive immunity via activation of the hypothalamic–pituitary–adrenal (HPA) axis and sympathetic nervous system, collectively constituting SIDS (27–29). This immune suppression impairs mucosal defense and phagocytic clearance, creating a permissive milieu for pulmonary infection (9, 30). PLR, by simultaneously capturing both arms of this dysregulation — innate overactivation and adaptive suppression — serves as a mechanistically coherent composite surrogate of the immune imbalance that predisposes AIS patients to SAP (Table 5).

**Table 5:** Threshold effect and linear association between log_2_-transformed PLR and stroke-associated pneumonia.

SAP outcome	Adjusted OR (95% CI)	*p*-value
Model I: Linear association (log₂-PLR, per 1-unit increase)		
PLR (Model I)	**1.36 (1.12–1.65)**	**0.0021**
Model II: Two-piecewise logistic regression		
Inflection point (K) *log₂-PLR = 7.812 (PLR = 224.8)*	*95% CI: 5.755–7.895*	—
PLR < 224.8 (below K)	1.21 (0.93–1.57)	0.1551
PLR ≥ 224.8 (above K)	**2.06 (1.10–3.87)**	**0.0240**
Difference (Segment 2–1)	1.71 (0.87–3.38)	0.1228
Log-likelihood ratio test	—	0.1724

Multivariable logistic regression models evaluating the linear and two-piecewise associations between log₂-transformed PLR and the risk of stroke-associated pneumonia (SAP).

Model I assumes a linear effect (overall effect estimate). Model II incorporates a threshold at the inflection point (K = log2-PLR 7.812, corresponding to PLR = 224.8). The inflection point was identified as the cut-point yielding the maximum log-likelihood among candidate cut-points in the 10th–90th percentile range; the 95% CI was estimated via 200 bootstrap resamples.

Both models are adjusted for: Age, Gender, Smoke, Hypertension, Diabetes, Atrial fibrillation, COPD, FPG, HbA1C, TG, TC, UA, HCY, CRP, WBC, Albumin, eGFR, NIHSS, KWDT.

Segment ORs represent the association per 1-unit increase in log₂-PLR within each segment. The difference row tests whether the two slopes are significantly different (OR = exp(β₂−β₁)).

Bold values indicate statistical significance (*p* < 0.05). —, not applicable.

PLR, platelet-to-lymphocyte ratio; SAP, stroke-associated pneumonia; OR, odds ratio; CI, confidence interval; K, inflection point.

The threshold effect identified in our study, with SAP risk increasing above PLR = 224.8, may have clinical implications. The absence of a significant association below this threshold suggests that modest PLR elevations may have limited standalone value, whereas higher values may identify patients with more severe systemic inflammation or immune dysregulation. The threshold should be considered hypothesis-generating, because the between-segment comparison (*p* = 0.1228) and the likelihood ratio test (*p* = 0.1724) were not statistically significant.

The discriminatory performance of PLR alone (AUC = 0.584) was substantially inferior to CRP (AUC = 0.825) and A2DS2 (AUC = 0.757). The absence of a significant gain when PLR was combined with CRP or A2DS2 suggests overlapping predictive information with these established markers. The PLR-SAP association observed in regression models, despite modest standalone discrimination, is not contradictory: adjusted association and classification performance answer different clinical questions. PLR nevertheless remains readily available from the routine complete blood count and may be useful as an adjunct to early risk assessment.

Our findings align with and extend the growing literature on hematological inflammation indices in cerebrovascular disease. Several prior studies have investigated SII and NLR in AIS (10, 31–33), but PLR’s specific role in SAP has received limited attention. The threshold-dependent pattern we identified using GAM and piecewise regression contrasts with the predominantly linear analyses in prior work, and echoes threshold effects reported for SII in the same clinical context (31). The application of non-linear modeling is increasingly recognized as essential in biomarker research, given that biological systems often exhibit saturation or threshold behaviors rather than strictly linear dose–response relationships (34, 35). Two related studies from the same institution should be distinguished from independent validation. Duan et al. reported the C-reactive protein-to-albumin ratio in 1,595 patients admitted from September 2016 to September 2022 (36), whereas Zhang et al. reported the neutrophil-to-lymphocyte ratio in 1,473 afebrile patients admitted from January 2019 to December 2023 (37). The present PLR analysis uses the same source dataset and time window as the NLR study, and the patient source population therefore overlaps; however, the exact number of shared patients could not be calculated because no cross-study unique-identifier linkage file was available. The present analysis evaluates a distinct exposure and is not an external replication. The CAR study had a partly overlapping period but a different cohort size and eligibility/exclusion workflow; exact record-level overlap could not be quantified from the available historical data.

Several limitations should be acknowledged. First, the single-center retrospective design limits causal inference and generalizability; prospective multicenter validation is required. Second, PLR was measured within 24 h of admission, but the available data did not establish the exact temporal relationship between blood sampling and early radiographic changes, aspiration events, or antibiotic administration; reverse causation therefore cannot be excluded. Third, single imputation of missing HbA1c values may underestimate imputation uncertainty. Fourth, residual confounding by unmeasured variables, including infarct volume, lesion location, stroke etiology, witnessed aspiration events, and pre-admission antibiotic use, cannot be excluded. Possible overadjustment from including CRP and WBC in the fully adjusted model is also possible. Fifth, the PLR > 1,000 exclusion and the data-driven threshold may affect generalizability and require external validation. Internal validation indicated modest optimism, with the AUC decreasing from 0.906 to 0.897 after bootstrap correction; however, external validation in an independent cohort remains necessary before clinical implementation.

## 5 Conclusion

Elevated admission PLR is associated with SAP in AIS patients, with a possible non-linear relationship and higher risk above PLR = 224.8. The association was consistent across predefined clinical subgroups. However, PLR demonstrated modest standalone discrimination and did not add significant ROC improvement to CRP or A2DS2. PLR may therefore serve as an accessible adjunct for early SAP risk assessment, but the threshold and clinical utility require prospective multicenter validation.

## Data Availability

The original contributions presented in the study are included in the article/supplementary material and are available from the corresponding author upon reasonable request, subject to approval by the Institutional Ethics Committee.
