# Supplementary material for: Elevated platelet-to-lymphocyte ratio is associated with stroke-associated pneumonia in acute ischemic stroke: a retrospective cohort study
Source: Front Neurol. 2026 Oct 2;17:1891885. doi: 10.3389/fneur.2026.1891885 (PMC13671966; doi:10.3389/fneur.2026.1891885)
Supplement: Supplementary file 1 [file Table_1.docx]

**Supplementary Table S1. Variance inflation factors (VIF) for covariates included in the multivariable logistic regression model**

| **No.** | **Variable** | **VIF** | **Interpretation** |
| --- | --- | --- | --- |
| 1 | Age (years) | 1.690 | No collinearity |
| 2 | Temperature (°C) | 1.065 | No collinearity |
| 3 | Gender | 2.262 | Mild collinearity |
| 4 | Smoke | 1.728 | No collinearity |
| 5 | Hypertension | 1.074 | No collinearity |
| 6 | Diabetes | 1.930 | No collinearity |
| 7 | Atrial fibrillation | 1.542 | No collinearity |
| 8 | Nasal feeding | 4.014 | Mild collinearity |
| 9 | Disturbance of consciousness | 2.150 | Mild collinearity |
| 10 | COPD | 1.119 | No collinearity |
| 11 | FPG (mg/dL) | 2.712 | Mild collinearity |
| 12 | HbA1C (%) | 3.423 | Mild collinearity |
| 13 | TG (mg/dL) | 1.265 | No collinearity |
| 14 | TC (mmol/L) | **6.282** | Moderate collinearity |
| 15 | LDL-C (mmol/L) | **5.943** | Moderate collinearity |
| 16 | UA (μmol/L) | 1.366 | No collinearity |
| 17 | HCY (μmol/L) | 1.122 | No collinearity |
| 18 | CRP (mg/L) | 1.248 | No collinearity |
| 19 | WBC (×10⁹/L) | **8.341** | Moderate collinearity |
| 20 | NTC (×10⁹/L) | **8.726** | Moderate collinearity |
| 21 | Albumin (g/L) | 1.175 | No collinearity |
| 22 | eGFR (mL/min/1.73 m²) | 1.455 | No collinearity |
| 23 | NIHSS | **5.161** | Moderate collinearity |
| 24 | KWDT | **5.651** | Moderate collinearity |
| 25 | A2DS2 | **7.688** | Moderate collinearity |
| 26 | GCS | 2.757 | Mild collinearity |

*VIFs were calculated in the final analytic cohort (N = 1,745) after exclusion of the single record with missing eGFR and use of the single-imputed HbA1c values. SAP (outcome) and PLR (primary exposure) were excluded from the VIF analysis.*

*The current analysis dataset included HbA1c values imputed once using multiple imputation by chained equations with a random-forest estimator. VIF < 2 indicates no collinearity; 2 <= VIF < 5 indicates mild collinearity; 5 <= VIF < 10 indicates moderate collinearity; and VIF >= 10 indicates severe collinearity.*

*The maximum VIF was 8.726 for NTC (×10⁹/L); all VIF values were below 10, indicating no severe multicollinearity among the covariates.*

*Abbreviations: PLR, platelet-to-lymphocyte ratio; SAP, stroke-associated pneumonia; FPG, fasting plasma glucose; HbA1C, glycated hemoglobin; TG, triglycerides; TC, total cholesterol; LDL-C, low-density lipoprotein cholesterol; UA, uric acid; HCY, homocysteine; CRP, C-reactive protein; WBC, white blood cell count; NTC, neutrophil count; eGFR, estimated glomerular filtration rate; NIHSS, National Institutes of Health Stroke Scale; KWDT, Kubota Water Drinking Test; A2DS2, age, atrial fibrillation, dysphagia, sex, stroke severity; GCS, Glasgow Coma Scale.*

*Bold red VIF values (>= 5) indicate moderate collinearity; all remained below the critical threshold of 10.*
