# Supplementary material for: Elevated platelet-to-lymphocyte ratio is associated with stroke-associated pneumonia in acute ischemic stroke: a retrospective cohort study
Source: Front Neurol. 2026 Oct 2;17:1891885. doi: 10.3389/fneur.2026.1891885 (PMC13671966; doi:10.3389/fneur.2026.1891885)
Supplement: Supplementary file 2 [file Table_2.docx]

**Supplementary Table S2. Sensitivity analysis and influence diagnostics for extreme PLR values**

| **No.** | **Analysis dataset** | **N (SAP)** | **Model 3 OR (95% CI)** | **PLR AUC (95% CI)** |
| --- | --- | --- | --- | --- |
| 1 | Retaining all PLR values | 1,766 (376) | 1.45 (1.23-1.71), P<0.001 | 0.601 (0.566-0.636) |
| 2 | Primary cohort (PLR <=1,000) | 1,745 (359) | 1.36 (1.12-1.65), P=0.0021 | 0.584 (0.549-0.619) |
| 3 | Leave-one-extreme-observation-out | 21 analyses | OR range: 1.421-1.480 | - |

*Sensitivity analyses used the pre-exclusion imputed dataset (N = 1,766; SAP = 376) and the primary cohort after excluding PLR values >1,000 (N = 1,745; SAP = 359).*

*Model 3 was adjusted for age, gender, smoking, hypertension, diabetes, atrial fibrillation, COPD, FPG, HbA1c, TG, TC, UA, HCY, CRP, WBC, albumin, eGFR, NIHSS, and KWDT. HbA1c values were imputed once using MICE with a random-forest estimator.*

*The pre-exclusion dataset retained 21 PLR values >1,000; its maximum PLR was 35,563.38 (PLT = 303; LTC = 0.00852). The primary cohort had no PLR values >1,000; its maximum PLR was 973.51.*

*Influence diagnostics among the 21 extreme observations showed a maximum Cook's distance of 0.0062 and a maximum absolute DFBETA for log2-PLR of 0.247. No winsorization or truncation was applied; the primary analysis used the defined PLR >1,000 exclusion rule.*

*Abbreviations: PLR, platelet-to-lymphocyte ratio; SAP, stroke-associated pneumonia; OR, odds ratio; CI, confidence interval; AUC, area under the receiver operating characteristic curve; PLT, platelet count; LTC, lymphocyte count; FPG, fasting plasma glucose; HbA1c, glycated hemoglobin; TG, triglycerides; TC, total cholesterol; UA, uric acid; HCY, homocysteine; CRP, C-reactive protein; WBC, white blood cell count; eGFR, estimated glomerular filtration rate; NIHSS, National Institutes of Health Stroke Scale; KWDT, Kubota Water Drinking Test.*

*The leave-one-extreme-observation-out analysis was performed by refitting Model 3 after removing each PLR >1,000 observation in turn.*
