# Supplementary material for: Elevated platelet-to-lymphocyte ratio is associated with stroke-associated pneumonia in acute ischemic stroke: a retrospective cohort study
Source: Front Neurol. 2026 Oct 2;17:1891885. doi: 10.3389/fneur.2026.1891885 (PMC13671966; doi:10.3389/fneur.2026.1891885)
Supplement: Supplementary file 3 [file Table_3.docx]

**Supplementary Table S3. Internal validation, calibration, and clinical utility of the multivariable prediction model**

| **No.** | **Metric** | **Apparent** | **10-fold OOF** | **Bootstrap** |
| --- | --- | --- | --- | --- |
| 1 | AUC | 0.906 | 0.900 | 0.897 |
| 2 | Brier score | 0.0834 | 0.0861 | 0.0873 |
| 3 | Log loss | 0.2894 | 0.3004 | - |
| 4 | Calibration intercept | - | -0.068 | - |
| 5 | Calibration slope | - | 0.942 | - |
| 6 | Mean optimism (AUC / Brier) | - | - | 0.0090 / 0.0039 |
| 7 | OOF DCA threshold range | - | 0.05-0.50 | - |

*The analysis used the primary cleaned cohort (N = 1,745; SAP = 359) and the prespecified Model 3 covariates: age, gender, smoking, hypertension, diabetes, atrial fibrillation, COPD, FPG, HbA1c, TG, TC, UA, HCY, CRP, WBC, albumin, eGFR, baseline NIHSS, and KWDT.*

*Internal validation was performed using 10-fold out-of-fold (OOF) cross-validation and 1,000 bootstrap resamples. AUC and Brier score are reported as apparent, OOF, and bootstrap optimism-corrected estimates where available. Lower Brier score and log loss indicate better performance; a calibration intercept of 0 and slope of 1 indicate ideal calibration.*

*The OOF decision-curve analysis showed positive net benefit for the model and exceeded the treat-all and treat-none strategies over the examined threshold-probability range of 0.05-0.50. The threshold range was exploratory and was not interpreted as a validated clinical decision threshold.*

*External validation was not performed because an independent cohort was unavailable. The reported internal-validation estimates do not establish transportability or clinical utility in other settings.*

*Abbreviations: AUC, area under the receiver operating characteristic curve; OOF, out-of-fold; DCA, decision-curve analysis; Brier, Brier score; SAP, stroke-associated pneumonia; FPG, fasting plasma glucose; HbA1c, glycated hemoglobin; TG, triglycerides; TC, total cholesterol; UA, uric acid; HCY, homocysteine; CRP, C-reactive protein; WBC, white blood cell count; eGFR, estimated glomerular filtration rate; NIHSS, National Institutes of Health Stroke Scale; KWDT, Kubota Water Drinking Test.*
